# 

**Published:** 1890-03-08

**Authors:** 


### THE HOSPITAL

A WEEKLY JOURNAL OF
Science, Medicine, Nursing and Philanthropy.

SATURDAY, MARCH 8th, 1890.

**Physic, Law, and Drunkenness** - - - 351
**Words of Consolation.**—Repentance - - - 352
**The Hospital Problem.** By Mr. Henry C. Burdett. I.—Hospital Income and Expenditure - - - 353
**The History and Capabilities of Herbal Simples.** By W. T. Fernie, M.D. VI.—The Common Barberry—Sweet Basil - - - 354
**Everybody's Page** - - - 355
**The Hospital Workshop.**—XXII.—The Taunton and Somerset Hospital - - - 356
**The Doctor's Pulpit.**—A Sermon for the Kitchen - - - 357
**Notes and News** - - - 358
**Annotations.**—Alcohol in Hospitals—The Lady and the Doctor—Monkeys and "Tight Lacing"— - - - 360
**Passing Topics.**—Small Special Hospitals - - - 366
**Scraps and Gleanings** - - - 366
**The Practitioner's Mirror and Retrospect:**
Medicine—Dengue in Europe—Diagnosis and Treatment of the Several Forms of Rheumatism—Cutis Laxa—Cystinuria—Colour Blindness - - - 361
Surgery—Reduction of Strangulated Herniæ by Coughing—Cure of Flat-Foot by Massage—Evacuation of Vertebral and Psoas Abscesses—Treatment of Abscesses in Cavities with Rigid Walls—Paracentesis in Empyema—Iodoform in Articular Tuberculosis—Arrest of Erysipelas - - - 362
Therapeutics—Prevention of Vomiting - - - 364
Hygiene—Dust and Disease - - - 364
Skin Department—Scleroderma - - - 364
New Drugs, Appliances, and Things Medical—Artificial Limbs (Messrs. Ferris)—Paraffinum Molle - - - 364
Practitioner's Bookshelf—The Year Book of Treatment for 1890 - - - 365

### EXTRA SUPPLEMENT—THE NURSING MIRROR.

**En Passant.**—Exhibition of Nursing Uniforms—Short Items—The Glasgow Training Home—Thousands of Pounds for the National Pension Fund—Cheltenham District Nurses—Registration of Midwives—Worcester News—Nursing at Berwick - - - xciii
**The Story of A Sermon** - - - xciv
**Lectures** - - - xciv
**The Nurses' Bookshelf.**—A Nurse at the Front—Medical Electricity— - - - xcv
**Examination Questions.**—Prize Answer for February xcv
**Appointments** - - - xcvi
**Presentation** - - - xcvi
**Notes and Queries—Vacancies** - - - xcvi
**Amusements and Relaxation** - - - xcvi

